# A 90-Day Oral Toxicological Evaluation of the Methylurate Purine Alkaloid Theacrine

**DOI:** 10.1155/2016/6206859

**Published:** 2016-08-22

**Authors:** Amy Clewell, Gábor Hirka, Róbert Glávits, Philip A. Palmer, John R. Endres, Timothy S. Murbach, Tennille Marx, Ilona Pasics Szakonyiné

**Affiliations:** ^1^AIBMR Life Sciences, Inc., 2800 East Madison Street, Suite 202, Seattle, WA 98112, USA; ^2^Toxi-Coop Zrt., Magyar Jakobinusok tere 4/B, Budapest 1122, Hungary

## Abstract

A 90-day repeated-dose oral toxicological evaluation was conducted according to GLP and OECD guidelines on the methylurate purine alkaloid theacrine, which is found naturally in certain plants. Four groups of Hsd.Brl.Han Wistar rats (ten/sex/group) were administered theacrine by gavage doses of 0 (vehicle only), 180, 300, and 375 mg/kg bw/day. Two females and one male in the 300 and 375 mg/kg bw/day groups, respectively, died during the study. Histological examination revealed centrilobular hepatocellular necrosis as the probable cause of death. In 375 mg/kg bw/day males, slight reductions in body weight development, food consumption, and feed efficiency, decreased weight of the testes and epididymides and decreased intensity of spermatogenesis in the testes, lack or decreased amount of mature spermatozoa in the epididymides, and decreased amount of prostatic secretions were detected at the end of the three months. At 300 mg/kg bw/day, slight decreases in the weights of the testes and epididymides, along with decreased intensity of spermatogenesis in the testes, and lack or decreased amount of mature spermatozoa in the epididymides were detected in male animals. The NOAEL was considered to be 180 mg/kg bw/day, as at this dose there were no toxicologically relevant treatment-related findings in male or female animals.

## 1. Introduction

Theacrine (1,3,7,9-tetramethyluric acid) is a methylurate, which is a class of purine alkaloids similar in structure to methylxanthines such as caffeine. Theacrine is often found as a methylated and oxidized metabolite of caffeine in methylxanthine-producing plants [1]. The two prominent theacrine-containing foods in the human diet are the fruits and seeds of* Theobroma grandiflorum* (cupuaçu) and kucha green tea from the leaves of* Camellia kucha* (*Camellia assamica *var.* kucha*) [2–8]. Kucha tea leaves have historically been consumed in certain regions of China as a tea and “healthy beverage” [9–11]. The theacrine content of expanding buds and young leaves of kucha has been reported as ~2.8% of dry weight and the content of mature leaves as ~1.3% [2, 3, 11]. As an estimate of possible exposure to theacrine from kucha tea, if one were to assume 2-3 grams of tea is used per cup at a theacrine content of 2.8%, a cup of tea would contain approximately 56–84 mg of theacrine (equivalent to 0.8–1.2 mg/kg bw for a 70 kg person). Radiolabelled experiments show that theacrine is synthesized from caffeine in some plants including kucha [2, 4]. Levels of theacrine in cupuaçu plant parts are not well-characterized in the literature.

Only limited research, primarily in cell and animal lines, is available to highlight any potential impact theacrine may have when ingested by humans. Preliminary data from a seven-day oral repeated-dose study by Feduccia et al. [12] demonstrated that theacrine increased locomotor activity in rats while an older study showed a potential biphasic dose-response curve with regard to its effects on activity in mice [13]. Mechanistically, theacrine appears to have adenosine receptor antagonist activity [12]. Other reports have highlighted theacrine's potential to exert dopaminergic and other neurochemical activity suggesting dose-dependent anti-inflammatory, antifatigue, analgesic, and mood enhancing bioactivity, although studies in humans are lacking [14–16].

Theacrine exhibited hepatoprotective effects in a stress-induced liver damage mouse model as well as strong antioxidant capacity in vitro and in vivo [3, 17]. In opposition to effects typically seen with caffeine [18, 19], Feduccia et al. showed that intraperitoneal injections of up to 48 mg/kg theacrine did not induce sensitization or tolerance of its physiologic effect over the seven-day period of the study [12].

Few studies on the safety of theacrine were found in a comprehensive literature search. Brief results of an acute toxicity study in mice were published in which the authors calculated the LD_50_ of orally administered theacrine as 810.6 mg/kg bw (95% confidence interval 769.5–858.0 mg/kg bw) [14]. Similar to other purine alkaloids, theacrine was reported to induce chromosomal aberrations in onion root tips, in* Vicia faba* cells treated during the G2 stage of interphase and in Chinese hamster cells [20, 21]. However, no genotoxicity was found in an in vivo mouse micronucleus study at theacrine concentrations up to 325 mg/kg bw [22].

In contrast to results observed using* C. sinensis* (31 g/kg caffeine and 0 g/kg theacrine), intragastric administration of water extracts of theacrine-containing teas including* C. assamica* var.* kucha* (3 g/kg caffeine and 22 g/kg theacrine) did not lead to increases in blood pressure and heart rate in spontaneously hypertensive rats [10]. When rats were given 30 mg/kg caffeine, theobromine, or theacrine, only the caffeine treatment had a significant effect on cardiovascular parameters [10].

In a human study of 60 healthy men and women, theacrine was given daily (200 or 300 mg) for eight weeks [23, 24]. The two doses are equivalent to 2.6 and 3.8 mg/kg bw/day, respectively, for a 78 kg human (the average weight for male and female subjects in the study). Primary outcomes included fasting clinical safety markers (heart rate, blood pressure, lipid profiles, and hematologic and liver/kidney/immune function biomarkers), all of which fell within normal limits with no group × time interactions and no differences in side effect profiles as compared to controls. Theacrine was also given to 15 healthy subjects in a randomized double-blinded crossover study [25, 26]. A single 200 mg dose (or placebo) was administered, and side effect reports, hemodynamics, and biochemical markers of safety were collected over a 3-hour postdosing period, with no significant findings noted. Six subjects additionally participated in a separate 7-day open-label repeated-dose study comparing 100, 200, and 400 mg of theacrine, in which no side effects were noted [25, 26].

To investigate further the safety of oral consumption of theacrine, in the current work we report the results of a 90-day repeated-dose oral subchronic toxicity study in the Wistar rat.

## 2. Material and Methods

The 90-day study was conducted according to OECD GLP (ENV/MC/CHEM (98)17; OECD, Paris, 1998) and in compliance with OECD 408 (adopted 21st September 1998; 90-day study) [27] and* US FDA Redbook 2000*, IV.C.4.a (2003; 90-day study) guidelines [28]. Care and use of study animals were in compliance with the laboratory's Institutional Animal Care and Use Committee, the National Research Council Guide for Care and Use of Laboratory Animals [29], and the principles of the Hungarian Act 2011 CLVIII (modification of Hungarian Act 1998 XXVIII) regulating animal protection.

Synthetic 1,3,7,9-tetramethyluric acid (CAS number 2309-49-1; ≥98% pure as measured by high performance liquid chromatography (HPLC), proton nuclear magnetic resonance, and liquid chromatography-mass spectrometry methodologies) was supplied as the branded product TeaCrine® for use as the test article by its manufacturer (Compound Solutions, Inc., Carlsbad, CA). TeaCrine is a commercially available white crystalline powder. A 24-month stability study on this product was conducted at 25 ± 2°C with 60 ± 10% relative humidity under conditions of commercial packaging and the compound remained stable throughout the testing period (data not shown). Batch number 48-KY20141102, which met all commercial specifications for the product (including ≥98% purity, ≤1% loss on drying, ≤0.5% residue on ignition, and commercial limits for heavy metals and microbial counts) was utilized for the study within the two-year shelf-life date. The specific purity level of this batch (per HPLC analysis) was 99.5%.

The dose levels of theacrine utilized in the study were 375, 300, and 180 mg/kg bw/day. These doses were chosen based on an unpublished 14-day repeated-dose oral toxicity study in Wistar rats that utilized ten animals per group (five rats/sex/group). The highest dose group of 500 mg/kg bw/day resulted in mortality of 5 of 5 males and 3 of 5 females and tremors in all animals; additionally one male animal died in the 400 mg/kg bw/day group. Remaining animals in the 400, 350, and 200 mg/kg bw/day groups survived without toxicological signs, and the NOAEL of the 14-day study was determined to be 350 mg/kg bw/day. Based on the results of this study and OECD 408 guidelines stating that the highest dose level should be chosen with the aim to induce toxicity but not death or severe suffering, the high dose for the 90-day study was selected as 375 mg/kg bw/day. The guidelines suggest a descending dose sequence aiming to demonstrate any dose-related responses and a NOAEL at the lowest dose level. While the guidelines state that twofold to fourfold intervals are frequently optimal for setting descending dose levels, in this case smaller intervals were utilized due to the narrow dose range in which adverse events appeared in the 14-day study and with an aim to detect the highest NOAEL possible (which a broader interval may have missed) to allow assessment of the margin of safety of doses used in human studies, such as the 200–300 mg per day dose (2.6–3.8 mg/kg bw/day for a 78 kg human) used in the study by Taylor et al. [24], which did not result in adverse events or findings in clinical safety markers.

The test article doses were prepared by suspending theacrine in 1% aqueous methylcellulose to achieve concentrations of 18, 30, and 37.5 mg/mL in order to provide a constant dosing volume of 10 mL/kg bw. Doses were prepared daily by careful weight measurement and administered within four hours. The control group received the same volume of 1% methylcellulose vehicle only.

Male and female SPF Hsd.Brl.Han Wistar rats (Toxi-Coop, Budapest, Hungary) were housed individually, with a 12-hour light-dark cycle at 19–25°C and 30–70% relative humidity, in type II polypropylene/polycarbonate cages with Lignocel® certified laboratory wood bedding. Cages were 22 cm (width) by 32 cm (length) by 19 cm (height), and cages and bedding were changed weekly. Animals received ssniff® SM R/M-Z+H complete diet for rats and mice and potable tap water ad libitum. The animals were acclimated for seven days prior to the start of dosing.

At the start of the experimental period, animals were approximately seven weeks old and weighed 206–233 g (males) and 131–151 g (females). Eighty male and female rats were stratified by body weight and randomly assigned to four dose groups containing 10 rats/sex/group. Theacrine was administered by gavage daily each morning at doses of 0 (vehicle-control), 180, 300, or 375 mg/kg bw/day.

Animals were observed twice daily for morbidity and mortality. General cage-side observations for clinical signs were made on two occasions during the acclimation period and once daily during the dosing period, at approximately the same time each day, after administration of the test article. Detailed clinical observations were conducted once weekly, and a functional observational battery (FOB) was performed during the final week to assess parameters such as general physical condition and behavior, response to handling, sensory reactions to various stimuli, grip strength, and motor activity [30]. Measurements of body weight were conducted twice during the acclimation period, on the first experimental day prior to treatment, twice weekly during weeks 1–4, once a week during weeks 5–13, and immediately prior to sacrifice. Food intake was determined and food efficiency calculated once weekly. Ophthalmological examination was carried out on all animals prior to the experimental period and prior to study termination in control and high-dose group animals.

After an overnight fast (approximately 16 hours) following final administration of the test article, three blood samples were collected from the retroorbital venous plexus under Isofluran CP® (CP-Pharma Handelsgesellschaft GmbH, Germany) anesthesia (0.25 mL in tripotassium ethylenediaminetetraacetic acid tubes for hematology measurements, 1.0 mL in sodium citrate tubes for blood coagulation measurements, and 2.5 mL in serum separator tubes for clinical chemistry measurements) after which the animals were euthanized by exsanguination from the abdominal aorta. Blood samples were analyzed for hematologic [hematocrit (HCT), hemoglobin (HGB), red blood cell (RBC), white blood cell (WBC), white blood cell differential (neutrophils (NEU), lymphocytes (LYM), monocytes (MONO), eosinophils (EOS) and basophils (BASO)), platelet (PLT), mean corpuscular volume (MCV), mean corpuscular hemoglobin (MCH), mean corpuscular hemoglobin concentration (MCHC), and reticulocyte (RET)], blood coagulation (activated partial thromboplastin time and prothrombin time) and clinical chemistry [sodium (Na^+^), potassium (K^+^), glucose (GLUC), cholesterol, urea concentration, creatinine (CREA), total protein (TPROT), albumin (ALB), alanine aminotransferase (ALT), aspartate aminotransferase (AST), alkaline phosphatase (ALP), gamma glutamyl transferase (GGT), total bilirubin (TBIL), albumin/globulin ratio, bile acids, calcium (Ca^++^), chloride (Cl^−^), and inorganic phosphate (Pi)] parameters. Gross pathological examinations and determinations of selected absolute organ weights (liver, kidneys, adrenals, testes, epididymides, thymus, spleen, brain, heart, uterus with fallopian tubes, ovaries, and thyroid/parathyroid) were completed and relative organ weights (compared to body and brain weights) were calculated. Full histopathological examinations were conducted on the preserved organs and tissues (adrenals, aorta, bone marrow of the femur, brain (cerebrum, cerebellum, pons, and medulla oblongata), eyes, female mammary gland, gonads (testes with epididymides and ovaries), heart, kidney, large intestines, liver, lungs, submandibular and mesenteric lymph nodes, quadriceps muscle, esophagus, nasal turbinates, pancreas, pituitary, prostate, submandibular salivary glands, sciatic nerve, seminal vesicle, skin, small intestines, spinal cord at three levels, spleen, sternum, stomach, thymus, thyroid and parathyroid, trachea and urinary bladder, and uterus with vagina) of all animals of the control and high-dose groups. The adrenal glands, testes, and epididymides were also processed and examined histologically in all animals of the low- and mid-dose groups on the basis of the macroscopic observations at the necropsy (pale adrenal glands and smaller than normal testes and epididymides).

Statistical analyses were conducted using SPSS PC+ software (SPSS, Inc., Chicago, IL). Bartlett's homogeneity of variance test was used to assess heterogeneity of variance between groups and was followed by a one-way analysis of variance (ANOVA) if no significant heterogeneity was detected. Duncan's Multiple Range test was used to assess the significance of intergroup differences if a positive ANOVA result was obtained. Where significant heterogeneity was detected by Bartlett's test, the Kolmogorov-Smirnov test was performed to examine normally distributed data, and Kruskal-Wallis nonparametric one-way ANOVA, followed by the Mann-Whitney* U* test for intergroup comparisons of positive results, was used in the case of a nonnormal distribution. A *p* value of <0.05 was considered statistically significant, and statistically significant results were reported at *p* < 0.05 and *p* < 0.01 levels.

## 3. Results and Discussion

**Figure 1:**
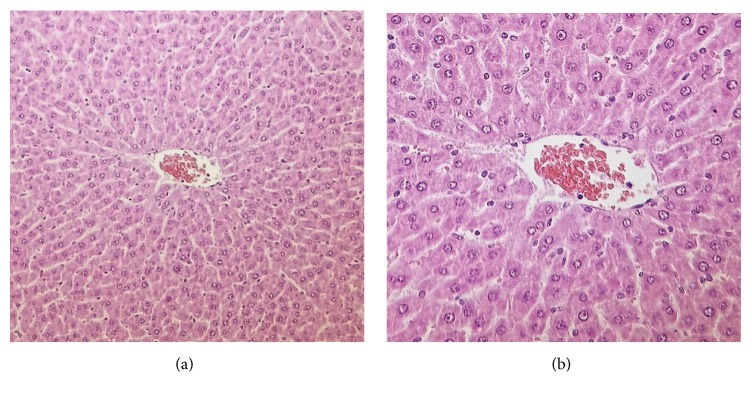
Intact (normal) hepatocytes around the central vein of a female rat at 375 mg/kg bw/day at terminal sacrifice. Haematoxylin and eosin staining; magnification 200x (a) and 400x (b).

**Figure 2:**
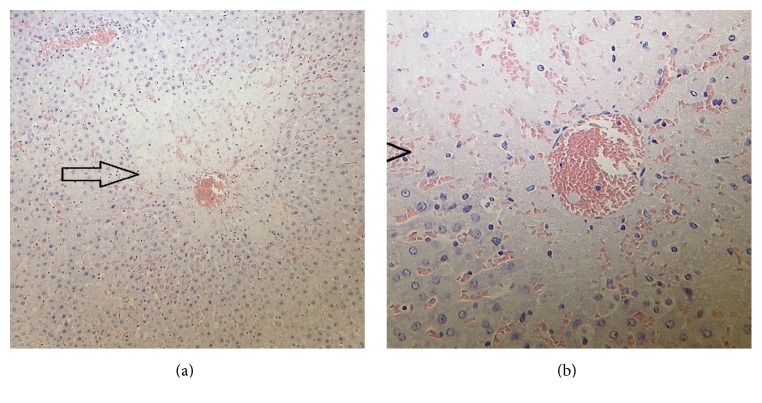
Centrilobular necrosis (arrows) in the liver of a female rat at 300 mg/kg bw/day found dead on day 33. Haematoxylin and eosin staining; magnification 200x (a) and 400x (b).

One male at 375 mg/kg bw/day and two females at 300 mg/kg bw/day were found dead on days 42, 33, and 67, respectively. There were no preceding clinical signs in the dead male and in one of the dead females. The other female exhibited a decrease in activity on the day before death. Necropsy observations of the dead animals revealed dark red liver (all) and lungs (male and one female), smaller than normal testes (male), clotted blood in the thoracic cavity near to the heart (male), cyanotic skin and subcutaneous connective tissue on the lower part of the abdomen (male), empty stomach (both females) and intestines (one female), hydrometra (one female), and enlarged adrenal glands (one female) (Table 1). During histopathological examination centrilobular hepatocellular necrosis was noted in all three animals (Figures 1 and 2). Chemically induced liver injury can lead to lipidosis, necrosis, fibrosis, and proliferation of organelles, hyperplasia of bile ducts or hepatocytes, and neoplasia [31]. Hepatocellular necrosis may be seen in aging and surviving untreated animals as well as those exposed to toxic chemicals. Necrosis may be coagulative in nature and characterized by homogenous eosinophilia and loss of cellular detail. Chemically induced necrosis is often zonal, most frequently centrilobular or periportal. Thus, the test article was considered to have most likely caused the centrilobular necrosis seen in these animals, and it was considered the probable cause of death.

Slight focal alveolar emphysema and congestion in the lungs and liver were also noted in the three dead animals and, along with the macroscopic changes in the lungs, liver, and heart and the cyanotic skin and subcutaneous connective tissue, were considered to have occurred due to circulatory disturbances developed during agony and/or death. Additionally, a decreased amount of spermatozoa in the epididymides and decreased intensity of spermatogenesis (defined by the proportion of tubuli containing mature spermatozoa) in the testes were observed in the male (Table 2).

In surviving animals, the daily cage-side and weekly detailed clinical observations and the FOB revealed no toxicologically relevant findings. A reduced body weight gain was detected in male and female animals in the 300 and 375 mg/kg bw/day groups and in male animals of the 180 mg/kg bw/day group between days 0 and 3 (Table 3). The reduced body weight gain of male animals in the 300 and 375 mg/kg bw/day groups resulted in lower mean body weight from day 3 to day 89 (Table 4) and lower mean total body weight gain with respect to controls. However, this reduced mean body weight gain on days 0 to 3 was fully compensated in male animals of the 180 mg/kg bw/day group and in both female groups (300 and 375 mg/kg bw/day) during the course of the treatment period resulting in no difference in the summarized mean body weight gain in these groups.

During week 1, food consumption was slightly decreased compared to controls in male and female treated animals in all dose groups and also occurred in male animals at 300 and 375 mg/kg bw/day on other weeks (Table 5). In accordance with the changes in body weight and food consumption, the mean feed efficiency was decreased in male animals at the 300 and 375 mg/kg bw/day dose levels during week 1 and transiently thereafter (Table 6).

No ophthalmologic abnormalities were observed in the control and 375 mg/kg bw/day groups prior to the start of dosing or at the end of the treatment period (data not shown). Statistically significant differences between treatment and controls were noted in some hematological and clinical chemistry parameters in male and female animals and are shown in Tables 7 and 8, respectively. Statistically significant differences in MCHC and RET values as compared to controls in males and RBC values in both males and females were not clearly dose-dependent and fell well within historical control ranges and were thus not considered toxicologically relevant. EOS values appeared to decrease dose-dependently within historical ranges in both genders; however, decreases in this value are not generally considered biologically relevant. Significant differences in MCV and MCH levels were slight and values remained within historical control ranges in the 180 mg/kg bw/day group (and were within or marginal to historical control ranges in the mid- and high-dose groups). Related hematological parameters such as HGB and HCT were not different than controls, and no hematologically related organ pathologies were noted. Thus the findings were not considered toxicologically adverse.

Slight but statistically significant increases were observed in liver ALT and AST enzyme activities in the 300 (ALT) and 375 (ALT and AST) mg/kg bw/day groups. Similarly, the mean CREA concentrations were slightly elevated in male treated animals. These slight, apparently dose-dependent changes may be indicative of a test article effect on hepatic and renal function; however, there were no related histopathological changes in the kidneys or livers of these animals to substantiate their relevance, and the values all remained well within historically normal ranges.

Interestingly, at lower doses in mice, theacrine (up to 30 mg/kg bw/day for seven days) was reported to protect against increases in ALT and AST levels induced by restraint stress [17]. Yet, in another recently published 90-day study, Crl: Sprague Dawley CD IGS rats given 150 mg/kg bw/day of the structurally similar compound, caffeine, also showed increases in AST, ALT, and CREA that fell within historical control ranges [32]. Significant differences in AST and ALT were reported in a National Toxicology Program study in Fischer 244 rats on caffeine at doses up to 287 mg/kg bw/day; however, no dose-related patterns were established [33]. Slight but significant increases in AST and ALT have also been reported in humans with consumption of coffee [34], although coffee/caffeine consumption has also been associated with protective effects against increases in liver enzymes (e.g., ALT) and liver protection in general [35–37]. Caffeine (and likely theacrine) is metabolized in the liver [33, 38] and thus high doses could theoretically have an effect on this organ due to high exposure chronically.

Other statistically significant differences in clinical chemistry values in various dose groups were slight and considered to be of little or no biological or toxicological relevance. For example, slight statistically significant differences in TBIL and K^+^, as compared to controls, occurred only in one gender, were not dose-dependent, and remained well within the historical control ranges. GLUC and Na^+^ values appeared to decrease statistically significantly and dose-dependently in both genders suggesting a possible test article effect, although all values remained well within historical control ranges. Differences in Cl^−^ and Pi did not show clear dose-response relationships.

Of note with regard to macroscopic findings (Table 1), smaller than normal testes (4/10 and 9/9) and epididymides (4/10 and 9/9) were observed in males of the 300 and 375 mg/kg bw/day groups, respectively. Three animals in the 375 mg/kg bw/day group also had smaller than normal prostates. Pale adrenal glands were observed in male animals at 300 mg/kg bw/day (2/10) and in male and female animals at 375 mg/kg bw/day (7/9 and 4/10, resp.). Other minor necropsy findings shown in Table 1 (e.g., white compact formation on the surface of the kidney, scarring, and alopecia in several groups) were considered to be individual findings in male animals as they are common observations in untreated experimental rats of this strain and age.

Decreased organ weights compared to controls were observed in male animals in the testes of the 300 (absolute and relative to brain weight) and 375 (absolute and relative to body and brain weights) mg/kg bw/day groups and epididymides of the 300 and 375 (absolute and relative to body and brain weights) mg/kg bw/day groups. Increased weights compared to controls were noted for adrenal glands in males at 375 (absolute and relative to body and brain weight) and 300 mg/kg bw/day (relative to body weight only). Decreases in thymus weight (absolute and relative to body and brain weight) were noted at 300 and 375 mg/kg bw/day in both male and female animals (Tables 9 [Table tab10]–11). Statistically significant differences in the weights of some organs in male animals (heart and kidneys) relative to body weight arose partially or fully from the body weight changes of these groups and were not seen in organ to brain weight ratios. Differences in some organ weights (absolute or relative) were observed only in the lower dose groups but not in the higher dose groups (liver, thyroid, and uterus) and, therefore, were not considered treatment-related.

In surviving animals, histological examination (Table 2) revealed decreased intensity of spermatogenesis in the seminiferous tubuli in all male animals at 375 mg/kg bw/day and in half of male animals at 300 mg/kg bw/day as compared to controls. In all animals with testicular findings, giant cells in the seminiferous tubuli were noted. Lack of mature spermatozoa in the ductuli of epididymides (2/10 at 300 mg/kg bw/day and 8/9 at 375 mg/kg bw/day to a moderate or severe degree) and decreased number of mature spermatozoa (1/10 at 300 mg/kg bw/day and 1/9 at 375 mg/kg bw/day in minimal or mild degree) were seen in male animals. The alterations in the testes and epididymides were not accompanied by inflammation, degeneration, or necrosis. The number and cytomorphology of interstitial testicular cells were the same as in control male animals. A decreased amount of secretion in the tubuli of the prostate was observed in three male animals at 375 mg/kg bw/day. In the remaining male animals of the 300 mg/kg bw/day group (5/10) and in all animals of the 180 mg/kg bw/day and control groups, the various spermatogenic cells (spermatogonia, spermatocytes, spermatids, and spermatozoa)—representing different phases in the development and differentiation of the spermatozoons—and the interstitial cells appeared normal. Similar effects have been reported in rats after consumption of high levels of the purine alkaloids theobromine and caffeine, namely, atrophy of the testes and epididymides and spermatogenic cell degeneration, although the mechanism by which this occurs is unknown [39, 40]. However in human studies, caffeine intake has not been associated with adverse effects related to semen quality, and fertility levels have, overall, not consistently been linked to caffeine intake [41].

There were no other treatment-related findings upon microscopic examination of the selected tissues. Findings that were not considered toxicologically relevant occurred in a few animals; for example, slight, focal alveolar emphysema was observed in the lungs of some male and female animals in control and high-dose groups with similar incidence. This finding is connected to hypoxia, dyspnea, and circulatory disturbance that occurs during exsanguination and was considered unrelated to test article administration [42]. Hyperplasia of bronchus-associated lymphoid tissue (BALT) was also observed in both the control and high-dose groups (with greater incidence in the control group). This is a physiological, immunomorphological phenomenon [43, 44] and is not considered toxicologically relevant. Dilatation of the uterine horns occurred in female animals of control and high-dose groups; this is considered a slight neurohormonal phenomenon connected to the estrus phase of the inner genital organs and not toxicologically relevant [45].

No histopathological findings were noted in the adrenal or thymus glands. Thus the pale adrenals and differences in organ weights of the adrenals and thymus were considered likely to be an indication of the adaptive process (the response of the organ to environmental variation in order to maintain function/survival) or stress response, and the toxicological significance was considered equivocal as has also been seen with theobromine and caffeine consumption in rats [32, 39, 46, 47].

## 4. Conclusion

In summary, doses of up to 300 mg (3.8 mg/kg bw/day for a 78 kg human) given to healthy males and females in a previous clinical study did not result in any adverse effects or potential toxicological findings in numerous clinical safety markers [24]. In the present GLP and OECD 408 compliant toxicological study in Wistar rats, theacrine consumption was associated with mortality at 300 mg/kg bw/day in two of ten females and, at 375 mg/kg bw/day in one of ten males, with centrilobular hepatocellular necrosis considered the likely cause of death. Males in the 375 mg/kg bw/day group also had reductions in body weight gain, food consumption and feed efficiency, and decreased weight of the testes and epididymides, along with decreased intensity of spermatogenesis, amount of mature spermatozoa, and prostate secretions. Males of the 300 mg/kg bw/day similarly had decreased weight of testes and epididymides and decreased intensity of spermatogenesis and amount of mature spermatozoa. Based on observations made in this 90-day repeated-dose gavage toxicity study and the lack of toxicologically relevant findings in the low dose group, the NOAEL for theacrine is considered to be 180 mg/kg bw/day in male and female Wistar rats.

## Figures and Tables

**Table 1:** Summary of necropsy findings.

Organ	Observations^*∗*^	Males (mg/kg bw/d)					Females (mg/kg bw/d)				
		Control	180	300	375		Control	180	300		375
					Died early	Survivors			Died early	Survivors	
	No macroscopic findings	10/10	9/10	6/10	0/1	0/9	9/10	6/10	0/2	6/8	3/10
Testes	Smaller than normal	0/10	0/10	4/10	1/1	9/9	/	/	/	/	/
Epididymides	Smaller than normal	0/10	0/10	4/10	0/1	9/9	/	/	/	/	/
Prostate	Smaller than normal	0/10	0/10	0/10	0/1	3/9	/	/	/	/	/
Adrenal glands	Pale	0/10	0/10	2/10	0/1	7/9	0/10	0/10	0/2	0/8	4/10
	Enlarged	0/10	0/10	0/10	0/1	0/9	0/10	0/10	1/2	0/8	0/10
Kidney (left side)	White compact formation on the surface	0/10	0/10	0/10	0/1	1/9	0/10	0/10	0/2	0/8	0/10
Skin	Alopecia	0/10	1/10	1/10	0/1	0/9	0/10	0/10	0/2	0/8	0/10
	Scar	0/10	0/10	1/10	0/1	2/9	0/10	0/10	0/2	0/8	0/10
	Cyanotic	0/10	0/10	0/10	1/1	0/9	0/10	0/10	0/2	0/8	0/10
Liver	Dark red	0/10	0/10	0/10	1/1	0/9	0/10	0/10	2/2	0/8	0/10
Lungs	Dark red	0/10	0/10	0/10	1/1	0/9	0/10	0/10	1/2	0/8	0/10
Thoracic cavity	Clotted blood near to the heart	0/10	0/10	0/10	1/1	0/9	0/10	0/10	0/2	0/8	0/10
Stomach	Empty	0/10	0/10	0/10	0/1	0/9	0/10	0/10	2/2	0/8	0/10
Intestines	Empty	0/10	0/10	0/10	0/1	0/9	0/10	0/10	1/2	0/8	0/10
Uterus	Hydrometra	/	/	/	/	/	1/10	4/10	1/2	2/8	4/10

^*∗*^Number of animals with observations/number of animals examined.

/, not examined; mg/kg bw/day, milligrams per kilogram body weight per day.

**Table 2:** Summary of notable histopathology findings.

Organs	Observations^*∗*^	Incidence of observations per group Dose groups (mg/kd bw/d)					Incidence of observations per group Dose groups (mg/kd bw/d)				
		Control	180	300	375		Control	180	300		375
					Survivors	Died early			Survivors	Died early	
		*Males*					*Females*				
Epididymides	Decreased amount of spermatozoa	0/10	0/10	1/10	1/9	1/1	/	/	/	/	/
	Lack of spermatozoa	0/10	0/10	2/10	8/9	0/1	/	/	/	/	/
Testes	Decreased intensity of spermatogenesis	0/10	0/10	5/10	9/9	1/1	/	/	/	/	/
Liver	Congestion	0/10	/	/	0/9	1/1	0/10	/	/	2/2	0/10
	Centrilobular necrosis	0/10	/	/	0/9	1/1	0/10	/	/	2/2	0/10
Lungs	Alveolar emphysema	2/10	/	/	1/9	1/1	2/10	/	/	2/2	2/10
	Hyperplasia of BALT	2/10	/	/	1/9	0/1	1/10	/	/	0/2	0/10
	Congestion	0/10	/	/	0/9	1/1	0/10	/	/	2/2	0/10
Prostate	Decreased amount of secretion	0/10	/	/	3/9	0/1	/	/	/	/	/
Skin	Exudative dermatitis	0/10	1/1	1/1	2/9	0/1	0/10	0/10	0/10	0/10	0/10
Uterus	Dilatation	/	/	/	/	/	1/10	/	/	0/2	4/9

^*∗*^Number of animals with observations/number of animals examined.

/, not examined; BALT, bronchus associated lymphoid tissue; mg/kg bw/d, milligrams per kilogram body weight per day.

**Table 3:** Summary of mean body weight gain.

Group (mg/kg bw/d)		Body weight gain (g) between days																	Sum
		0–3	3–7	7–10	10–14	14–17	17–21	21–24	24–28	28–35	35–42	42–49	49–56	56–63	63–70	70–77	77–84	84–89	0–89
Males																			
Control	Mean	21.2	23.8	6.7	19.8	10.4	17.4	8.3	13.4	14.4	18.1	16.0	12.8	7.8	10.1	8.7	5.1	1.0	215.0
	SD	1.8	3.3	2.9	3.4	2.4	3.8	4.2	3.9	2.9	3.5	4.0	2.1	2.3	3.0	3.9	3.5	2.4	15.1
180	Mean	16.5	23.1	10.5	18.0	9.5	16.2	9.9	12.2	19.3	16.3	14.6	15.2	10.9	8.5	9.4	7.1	3.6	220.8
	SD	5.2	3.9	2.5	3.8	3.8	3.4	4.7	3.6	6.2	3.9	4.7	4.1	3.3	4.1	3.1	3.6	3.7	22.9
	SS	*∗∗*																	
300	Mean	10.3	21.6	7.7	16.9	8.4	13.2	10.6	9.8	16.1	13.3	14.3	12.1	9.7	10.6	11.1	0.9	4.9	191.5
	SD	7.1	4.6	4.5	6.6	3.4	2.6	4.2	3.2	4.5	9.8	3.6	2.6	3.1	3.6	2.5	8.3	6.2	30.5
	SS	*∗∗*					*∗*											*∗*	*∗*
375	Mean	−1.7	22.7	10.0	15.9	10.5	15.3	9.3	9.4	11.9	12.1	12.3	10.9	7.0	9.8	10.8	5.2	7.9	181.9
	SD	12.5	3.6	5.2	6.0	3.8	3.1	4.2	8.0	6.2	5.7	6.9	6.2	5.5	4.2	5.3	6.1	2.0	27.7
	SS	*∗∗*									*∗*							*∗∗*	*∗∗*
	*n*	10	10	10	10	10	10	10	10	10	10	9	9	9	9	9	9	9	9

Females																			
Control	Mean	10.9	13.0	6.9	9.4	5.2	7.0	4.4	6.2	11.6	6.1	5.9	6.5	0.7	5.6	5.7	3.8	2.2	111.1
	SD	3.7	1.6	3.4	2.3	2.8	1.6	3.4	2.7	4.2	3.6	3.8	2.1	3.6	2.8	4.2	4.1	3.3	13.7
180	Mean	9.1	13.1	9.0	8.3	5.7	8.8	3.6	8.2	9.7	5.3	7.5	7.3	1.1	4.2	4.5	4.6	4.0	114.0
	SD	3.1	3.7	4.6	2.0	5.1	4.2	2.0	3.8	4.4	3.8	4.6	2.7	2.8	3.4	4.6	5.1	3.8	14.5
300	Mean	6.7	14.3	7.8	8.8	6.7	9.1	4.5	8.7	9.6	8.3	8.0	4.3	7.2	2.1	8.1	2.4	2.4	118.5
	SD	5.1	2.8	3.6	4.7	3.4	2.2	3.4	2.1	2.5	2.5	4.5	4.2	2.5	3.6	2.4	3.2	1.8	16.5
	*n*	10	10	10	10	10	10	10	10	9	9	9	9	9	8	8	8	8	8
	SS	*∗*												*∗∗*					
375	Mean	5.0	12.8	10.0	12.3	6.1	6.4	6.5	7.9	9.5	7.1	9.2	4.7	4.5	3.5	6.6	3.2	5.2	120.5
	SD	2.5	2.7	3.5	3.3	4.4	4.4	4.3	3.3	5.2	7.6	5.2	2.4	2.5	3.9	5.0	4.5	3.8	21.4
	SS	*∗∗*												*∗∗*					

mg/kg bw/d, milligrams per kilogram body weight per day; SD, standard deviation; g, grams; SS, statistical significance; *n*, number of animals.

^*∗*^ *p* < 0.05; ^*∗∗*^ *p* < 0.01. *n* = 10 unless otherwise stated.

**Table 4:** Summary of mean body weight.

Group (mg/kg bw/d)		Body weight (g) on days																	
		0	3	7	10	14	17	21	24	28	35	42	49	56	63	70	77	84	89
Males																			
Control	Mean	218.6	239.8	263.6	270.3	290.1	300.5	317.9	326.2	339.6	354.0	372.1	388.1	400.9	408.7	418.8	427.5	432.6	433.6
	SD	7.2	7.9	10.0	11.2	12.9	13.9	16.8	15.6	16.8	17.0	18.2	19.5	18.8	18.4	18.8	18.1	19.7	18.9
	*n*	10	10	10	10	10	10	10	10	10	10	10	10	10	10	10	10	10	10
180	Mean	218.3	234.8	257.9	268.4	286.4	295.9	312.1	322.0	334.2	353.5	369.8	384.4	399.6	410.5	419.0	428.4	435.5	439.1
	SD	7.2	10.9	12.0	12.2	15.5	17.5	15.7	16.0	15.9	18.4	19.4	21.7	21.5	22.1	23.8	24.0	26.1	26.2
	±%	0	−2	−2	−1	−1	−2	−2	−1	−2	0	−1	−1	0	0	0	0	1	1
	*n*	10	10	10	10	10	10	10	10	10	10	10	10	10	10	10	10	10	10
300	Mean	217.2	227.5	249.1	256.8	273.7	282.1	295.3	305.9	315.7	331.8	345.1	359.4	371.5	381.2	391.8	402.9	403.8	408.7
	SD	6.2	9.3	12.0	12.5	14.8	14.8	14.8	16.9	16.9	18.0	23.9	25.2	25.5	26.0	27.0	27.3	28.6	31.7
	±%	−1	−5	−6	−5	−6	−6	−7	−6	−7	−6	−7	−7	−7	−7	−6	−6	−7	−6
	SS		*∗*	*∗*	*∗*	*∗*	*∗*	*∗∗*	*∗*	*∗∗*	*∗∗*	*∗∗*	*∗∗*	*∗∗*	*∗∗*	*∗*	*∗*	*∗*	*∗*
	*n*	10	10	10	10	10	10	10	10	10	10	10	10	10	10	10	10	10	10
375	Mean	217.1	215.4	238.1	248.1	264.0	274.5	289.8	299.1	308.5	320.4	332.5	348.2	359.1	366.1	375.9	386.7	391.9	399.8
	SD	5.5	13.4	12.0	11.0	13.3	14.3	14.8	15.9	17.1	16.4	18.3	16.7	20.0	22.9	23.7	25.1	28.2	28.4
	±%	−1	−10	−10	−8	−9	−9	−9	−8	−9	−9	−11	−10	−10	−10	−10	−10	−9	−8
	SS		*∗∗*	*∗∗*	*∗∗*	*∗∗*	*∗∗*	*∗∗*	*∗∗*	*∗∗*	*∗∗*	*∗∗*	*∗∗*	*∗∗*	*∗∗*	*∗∗*	*∗∗*	*∗∗*	*∗*
	*n*	10	10	10	10	10	10	10	10	10	10	10	9	9	9	9	9	9	9

Females																			
Control	Mean	139.8	150.7	163.7	170.6	180.0	185.2	192.2	196.6	202.8	214.4	220.5	226.4	232.9	233.6	239.2	244.9	248.7	250.9
	SD	5.5	6.7	7.4	8.8	10.2	11.1	12.0	11.5	13.0	12.0	13.4	13.8	14.3	14.2	14.2	16.2	15.5	17.2
	*n*	10	10	10	10	10	10	10	10	10	10	10	10	10	10	10	10	10	10
180	Mean	140.1	149.2	162.3	171.3	179.6	185.3	194.1	197.7	205.9	215.6	220.9	228.4	235.7	236.8	241.0	245.5	250.1	254.1
	SD	5.9	6.1	4.5	5.0	4.8	6.3	8.2	8.4	9.7	11.4	11.9	13.3	14.0	13.9	14.0	14.0	15.6	15.8
	±%	0	−1	−1	0	0	0	1	1	2	1	0	1	1	1	1	0	1	1
	*n*	10	10	10	10	10	10	10	10	10	10	10	10	10	10	10	10	10	10
300	Mean	139.4	146.1	160.4	168.2	177.0	183.7	192.8	197.3	206.0	216.4	224.8	232.8	237.1	244.3	245.4	253.5	255.9	258.3
	SD	2.0	4.4	4.6	5.8	7.3	7.2	8.4	9.9	9.8	10.8	11.4	13.3	14.5	13.4	15.5	15.7	16.1	17.1
	±%	0	−3	−2	−1	−2	−1	0	0	2	1	2	3	2	5	3	4	3	3
	*n*	10	10	10	10	10	10	10	10	10	9	9	9	9	9	8	8	8	8
375	Mean	141.0	146.0	158.8	168.8	181.1	187.2	193.6	200.1	208.0	217.5	224.6	233.8	238.5	243.0	246.5	253.1	256.3	261.5
	SD	5.2	6.1	6.9	7.6	8.8	7.9	10.5	11.7	11.2	15.9	16.8	16.2	16.5	16.0	19.1	21.1	21.5	23.4
	±%	1	−3	−3	−1	1	1	1	2	3	1	2	3	2	4	3	3	3	4
	*n*	10	10	10	10	10	10	10	10	10	10	10	10	10	10	10	10	10	10

±%, percent deviation versus control; mg/kg bw/d, milligrams per kilogram body weight per day; SD, standard deviation; g, grams; SS, statistical significance; *n*, number of animals.

^*∗*^ *p* < 0.05; ^*∗∗*^ *p* < 0.01.

**Table 5:** Summary of food consumption.

Group (mg/kg bw/d)		Daily mean food consumption (g/animal/day) on weeks												
		1	2	3	4	5	6	7	8	9	10	11	12	13
Males														
Control	Mean	25.0	25.2	26.4	26.9	25.8	25.9	26.9	25.7	24.2	25.3	23.8	25.4	24.1
	SD	1.56	1.76	1.93	1.74	2.13	1.94	2.25	1.87	2.04	2.50	2.03	2.31	2.47
180	Mean	23.1	25.3	26.6	27.2	27.1	26.8	26.9	26.1	25.3	26.7	25.3	26.2	26.5
	SD	1.52	1.70	2.13	1.82	1.88	2.16	2.27	2.24	1.88	1.65	1.45	1.59	1.84
	±%	−7.7	0.4	0.9	1.1	5.1	3.3	0.1	1.7	4.8	5.9	6.3	3.2	10.2
	SS	*∗*												*∗*
300	Mean	21.0	23.6	23.6	24.9	24.5	24.4	24.9	23.9	23.7	24.8	23.7	24.1	24.1
	SD	1.72	1.43	2.52	1.22	1.16	1.40	1.69	1.47	1.40	1.23	1.47	1.93	2.21
	±%	−16	−6	−11	−7	−5	−6	−7	−7	−2	−2	0	−5	0
	SS	*∗∗*		*∗∗*	*∗∗*			*∗*	*∗*					
375	Mean	18.2	22.8	24.4	24.7	23.2	23.2	24.4	23.2	22.6	23.8	23.2	24.6	24.7
	SD	2.76	2.48	1.62	1.43	1.86	1.71	1.54	1.94	1.86	2.04	2.15	2.76	2.21
	*n*	10	10	10	10	10	10	9	9	9	9	9	9	9
	±%	−27	−10	−8	−8	−10	−11	−9	−9	−7	−6	−2	−3	3
	SS	*∗∗*	*∗∗*	*∗*	*∗∗*	*∗∗*	*∗∗*	*∗*	*∗*					

Females														
Control	Mean	17.3	17.6	17.8	18.9	19.5	19.3	19.7	19.0	17.9	19.4	18.6	20.5	20.0
	SD	1.04	1.35	1.19	1.34	1.60	1.37	1.54	2.19	2.21	2.15	2.66	2.31	2.69
180	Mean	15.5	17.0	17.8	18.2	18.3	18.4	19.3	17.9	17.3	18.8	17.6	19.2	19.4
	SD	0.53	0.88	1.13	1.10	1.48	1.46	1.51	1.16	1.14	1.63	1.18	1.48	1.69
	±%	−10	−4	0	−4	−6	−5	−2	−6	−3	−3	−5	−6	−3
	SS	*∗∗*												
		*∗∗*												
300	Mean	14.6	16.5	17.8	18.4	18.4	18.7	19.5	18.1	17.8	18.8	18.9	20.5	19.6
	SD	1.81	1.13	1.41	1.12	1.39	0.98	1.61	1.46	1.03	0.68	2.83	3.47	1.18
	*n*	10	10	10	10	9	9	9	9	9	8	8	8	8
	±%	−15	−6	0	−3	−6	−3	−1	−5	0	−3	2	0	−2
	SS	*∗∗*												
		*∗∗*												
375	Mean	14.4	17.5	17.6	18.8	18.7	18.6	19.6	18.7	17.7	18.9	18.2	19.8	19.9
	SD	1.49	1.08	2.14	1.17	1.80	2.26	1.57	1.55	1.45	1.64	1.79	1.91	1.95
	±%	−16.9	−0.6	−1.0	−0.5	−4.2	−3.9	−0.3	−1.7	−1.2	−2.9	−2.1	−3.3	−0.2
	SS	*∗∗*												

±%, percent deviation versus control; mg/kg bw/d, milligrams per kilogram body weight per day; SD, standard deviation; g, grams; SS, statistical significance; *n*, number of animals.

^*∗*^ *p* < 0.05; ^*∗∗*^ *p* < 0.01. *n* = 10 unless otherwise stated.

**Table 6:** Summary of feed efficiency.

Group (mg/kg bw/d)		Feed efficiency (g bw/g food)													Sum
	Days	0–7	7–14	14–21	21–28	28–35	35–42	42–49	49–56	56–63	63–70	70–77	77–84	84–89	0–89
	Weeks	1	2	3	4	5	6	7	8	9	10	11	12	13	1–13
Males															
Control	Mean	0.26	0.15	0.15	0.12	0.08	0.10	0.09	0.07	0.05	0.06	0.05	0.03	0.02	0.10
	SD	0.02	0.02	0.02	0.02	0.01	0.02	0.02	0.02	0.01	0.01	0.02	0.02	0.02	0.01
180	Mean	0.24	0.16	0.14	0.12	0.10	0.09	0.08	0.08	0.06	0.04	0.05	0.04	0.04	0.09
	SD	0.03	0.01	0.02	0.03	0.03	0.02	0.02	0.02	0.02	0.02	0.02	0.02	0.01	0.01
	SS													*∗*	
300	Mean	0.21	0.15	0.13	0.12	0.09	0.09	0.08	0.07	0.06	0.06	0.07	0.02	0.05	0.09
	SD	0.05	0.05	0.03	0.03	0.02	0.02	0.02	0.01	0.02	0.02	0.02	0.02	0.04	0.01
	SS	*∗*												*∗*	
375	Mean	0.15	0.14	0.14	0.11	0.08	0.07	0.07	0.07	0.05	0.06	0.07	0.04	0.06	0.09
	SD	0.08	0.07	0.05	0.05	0.03	0.03	0.04	0.04	0.03	0.02	0.03	0.02	0.02	0.01
	SS	*∗∗*					*∗*							*∗∗*	

Females															
Control	Mean	0.20	0.13	0.10	0.08	0.08	0.05	0.05	0.05	0.02	0.05	0.05	0.03	0.04	0.07
	SD	0.02	0.02	0.02	0.03	0.03	0.02	0.02	0.02	0.02	0.01	0.03	0.02	0.02	0.01
180	Mean	0.20	0.15	0.11	0.09	0.07	0.04	0.05	0.06	0.02	0.04	0.05	0.05	0.06	0.07
	SD	0.04	0.04	0.04	0.03	0.03	0.03	0.03	0.02	0.01	0.02	0.02	0.03	0.03	0.01
	SS														
300	Mean	0.20	0.14	0.13	0.10	0.07	0.06	0.06	0.04	0.06	0.02	0.06	0.03	0.03	0.07
	SD	0.04	0.04	0.03	0.03	0.02	0.02	0.03	0.02	0.02	0.02	0.02	0.02	0.02	0.01
	SS									*∗∗*	*∗*				
375	Mean	0.18	0.18	0.10	0.11	0.08	0.07	0.07	0.04	0.04	0.04	0.06	0.04	0.07	0.07
	SD	0.02	0.04	0.04	0.03	0.03	0.02	0.04	0.02	0.02	0.01	0.03	0.02	0.03	0.01
	SS		*∗∗*												*∗*

±%, percent deviation versus control; mg/kg bw/d, milligrams per kilogram body weight per day; g bw/g food, grams body weight per grams of food; SD, standard deviation; g, grams; SS, statistical significance; *n*, number of animals.

^*∗*^ *p* < 0.05; ^*∗∗*^ *p* < 0.01.

**Table 7:** Summary of statistically significant hematological findings.

	Dose group (mg/kg bw/d)									
	Males					Females				
	Control *n* = 10	180 *n* = 10	300 *n* = 10	375 *n* = 9	Historical range	Control *n* = 10	180 *n* = 10	300 *n* = 8	375 *n* = 10	Historical range
WBC (×10^9^/L)	7.91 ± 0.97	7.69 ± 1.49	6.69 ± 1.79	8.13 ± 1.65	4.60–13.86	6.20 ± 1.81	5.63 ± 1.02	^*∗*^4.74 ± 0.98	6.57 ± 1.66	2.96–12.94
MONO (%)	2.82 ± 0.48	^*∗*^3.70 ± 0.85	2.62 ± 1.17	2.67 ± 0.68	0.6–4.1	2.09 ± 0.64	2.26 ± 0.31	2.51 ± 0.45	1.81 ± 0.48	0.4–3.1
EOS (%)	1.41 ± 0.40	^*∗*^1.04 ± 0.34	^*∗∗*^0.78 ± 0.20	^*∗∗*^0.69 ± 0.31	0.4–3.3	1.06 ± 0.43	0.78 ± 0.34	0.76 ± 0.32	^*∗∗*^0.52 ± 0.20	0.5–2.0
RBC (×10^12^/L)	9.51 ± 0.40	^*∗∗*^8.43 ± 0.48	^*∗∗*^8.31 ± 0.64	^*∗∗*^8.34 ± 0.33	6.20–10.26	8.67 ± 0.28	^*∗∗*^7.92 ± 0.40	^*∗∗*^8.01 ± 0.52	^*∗*^8.19 ± 0.67	7.61–9.31
HGB (g/L)	168.70 ± 5.93	162.00 ± 7.47	161.90 ± 10.84	163.67 ± 3.50	109–184	161.20 ± 3.77	^*∗*^154.60 ± 6.82	158.88 ± 9.49	165.20 ± 6.99	145–169
MCV (fL)	48.04 ± 1.40	^*∗∗*^52.96 ± 2.96	^*∗∗*^53.94 ± 2.15	^*∗∗*^54.17 ± 2.68	45.0–53.7	51.13 ± 2.09	^*∗∗*^54.18 ± 2.71	^*∗∗*^54.78 ± 2.15	^*∗∗*^55.14 ± 1.65	47.0–55.0
MCH (pg)	17.75 ± 0.41	^*∗∗*^19.24 ± 0.81	^*∗∗*^19.51 ± 0.57	^*∗∗*^19.66 ± 0.86	16.7–19.4	18.61 ± 0.62	^*∗*^19.54 ± 0.89	^*∗∗*^19.85 ± 0.56	^*∗∗*^20.25 ± 1.29	17.3–19.7
MCHC (g/L)	369.70 ± 4.40	^*∗*^363.70 ± 9.07	^*∗*^361.80 ± 5.92	^*∗*^362.89 ± 4.26	339–376	363.90 ± 5.90	360.60 ± 3.53	362.75 ± 5.50	367.20 ± 17.07	351–368
RET (%)	3.15 ± 0.30	^*∗∗*^3.67 ± 0.32	^*∗∗*^4.18 ± 1.72	^*∗∗*^3.94 ± 0.56	2.51–4.65	3.93 ± 0.49	4.14 ± 0.79	3.91 ± 0.63	4.36 ± 1.22	3.17–6.16

Values are expressed as mean ± standard deviation. Historical Range based on data from 40 male and 38 female Hsd.Brl.Han Wistar control rats aged 19-20 weeks.

mg/kg bw/d, milligrams per kilogram body weight per day; L, liter; fL, femtoliters; pg, picograms; *n*, number of animals; ^*∗*^ *p* < 0.05 and ^*∗∗*^ *p* < 0.01.

**Table 8:** Summary of statistically significant clinical chemistry findings.

	Dose group (mg/kg bw/d)									
	Males					Females				
	Control *n* = 10	180 *n* = 10	300 *n* = 10	375 *n* = 9	Historical range	Control *n* = 10	180 *n* = 10	300 *n* = 8	375 *n* = 10	Historical range
ALT (U/L)	54.97 ± 10.62	66.51 ± 7.29	^*∗∗*^73.55 ± 13.81	^*∗∗*^75.58 ± 18.09	38.5–98.0	58.26 ± 11.88	68.12 ± 12.14	^*∗*^73.925 ± 17.23	^*∗∗*^78.25 ± 14.91	31.4–84.1
AST (U/L)	94.64 ± 20.70	101.81 ± 12.75	98.25 ± 8.76	^*∗*^112.53 ± 17.90	72.6–123.5	95.25 ± 16.82	89.75 ± 16.20	99.39 ± 17.46	^*∗*^112.36 ± 20.00	75.0–116.6
ALP (U/L)	102.80 ± 20.47	^*∗∗*^78.8 ± 10.89	^*∗*^79.70 ± 12.28	89.44 ± 25.67	67–215	55.9 ± 17.25	72.90 ± 25.88	60.88 ± 35.45	73.40 ± 32.00	20–185
TBIL (*μ*mol/L)	1.71 ± 0.23	1.69 ± 0.28	^*∗*^2.07 ± 0.37	^*∗*^2.09 ± 0.35	0.62–4.72	1.839 ± 0.45	1.44 ± 0.34	1.86 ± 0.38	1.85 ± 0.62	0.91–4.69
CREA (*μ*mol/L)	28.70 ± 2.73	^*∗*^32.76 ± 3.97	^*∗∗*^34.35 ± 4.58	^*∗∗*^35.97 ± 2.89	23.1–37.0	29.3 ± 2.32	29.51 ± 3.28	30.30 ± 3.55	31.15 ± 3.06	25.9–38.2
Urea (*μ*mol/L)	7.48 ± 0.91	7.20 ± 0.88	7.64 ± 0.86	7.64 ± 0.82	4.14–9.62	6.947 ± 0.71	7.19 ± 1.04	6.83 ± 0.85	7.08 ± 0.82	4.79–9.71
GLUC (*μ*mol/L)	6.04 ± 0.52	5.99 ± 0.53	5.62 ± 0.43	^*∗∗*^5.35 ± 0.33	4.98–7.97	5.73 ± 0.49	5.71 ± 0.49	^*∗*^5.10 ± 0.80	^*∗∗*^4.53 ± 0.41	4.18–8.49
Pi (*μ*mol/L)	1.90 ± 0.28	2.09 ± 0.25	2.09 ± 0.20	^*∗∗*^2.39 ± 0.24	1.40–2.06	1.275 ± 0.16	^*∗*^1.53 ± 0.16	^*∗∗*^2.12 ± 0.20	^*∗∗*^2.11 ± 0.39	1.3–2.1
Ca^++^ (*μ*mol/L)	2.67 ± 0.08	^*∗*^2.58 ± 0.11	^*∗*^2.56 ± 0.09	2.63 ± 0.07	2.39–2.75	2.617 ± 0.06	2.55 ± 0.10	2.57 ± 0.07	2.54 ± 0.10	2.4–2.8
Na^+^ (*μ*mol/L)	142.00 ± 1.05	^*∗∗*^140.00 ± 1.49	^*∗∗*^138.80 ± 1.14	^*∗∗*^138.67 ± 0.87	137–147	140.6 ± 0.97	139.90 ± 1.79	139.25 ± 1.58	^*∗*^138.50 ± 2.51	137–147
K^+^ (*μ*mol/L)	4.41 ± 0.25	4.61 ± 0.39	4.23 ± 0.15	4.45 ± 0.26	3.62–5.31	3.921 ± 0.24	4.15 ± 0.21	^*∗*^4.28 ± 0.32	^*∗*^4.24 ± 0.45	3.6–4.4
Cl^−^ (*μ*mol/L)	105.65 ± 0.98	^*∗∗*^103.4 ± 1.70	^*∗∗*^102.24 ± 0.98	^*∗∗*^102.46 ± 1.34	101.0–106.6	104.66 ± 0.74	^*∗*^103.56 ± 1.37	^*∗∗*^102.79 ± 1.15	^*∗*^102.79 ± 2.22	101.2–109.6
ALB (g/L)	34.21 ± 0.72	^*∗*^33.08 ± 0.88	^*∗∗*^32.03 ± 1.61	33.63 ± 1.19	31.5–36.7	35.77 ± 1.29	34.73 ± 1.10	34.76 ± 1.45	35.03 ± 2.04	32.9–41.1
TPROT (g/L)	62.59 ± 3.30	^*∗*^59.7 ± 2.44	^*∗∗*^57.37 ± 2.30	61.22 ± 2.70	55.5–70.6	64.76 ± 3.53	62.09 ± 2.60	62.65 ± 2.66	64.29 ± 5.17	57.6–79.3

Values are expressed as mean ± standard deviation. Historical Range based on data from 40 male and 38 female (19 females for BUN and Pi) Hsd.Brl.Han Wistar control rats aged 19-20 weeks.

mg/kg bw/d, milligrams per kilogram body weight per day; U/L, units per liter; *μ*mol, micromol; g, gram; *n*, number of animals.

^*∗*^ *p*< 0.05; ^*∗∗*^ *p* < 0.01.

**Table 9:** Summary of organ weights.

Group (mg/kg bw/d)		Organ weight (g)										
		Body weight	Brain	Liver	Kidneys	Heart	Thymus	Spleen	Testes or uterus	Epididymides or ovaries	Adrenals	Thyroids
Males												
Control (*n* = 10)	Mean	422.0	2.16	10.00	2.28	1.14	0.44	0.71	3.49	1.62	0.077	0.028
	SD	17.52	0.16	0.96	0.20	0.09	0.12	0.10	0.34	0.09	0.01	0.01
180 (*n* = 10)	Mean	421.8	2.19	10.77	2.38	1.14	0.40	0.77	3.51	1.65	0.074	0.023
	SD	25.26	0.12	0.99	0.26	0.19	0.08	0.08	0.34	0.20	0.02	0.01
	±%	0.0	1	8	5	0	−8	9	1	2	−4	−19
	SS											*∗*
300 (*n* = 10)	Mean	393.1	2.11	9.83	2.33	1.20	0.32	0.80	2.70	1.36	0.085	0.021
	SD	28.39	0.10	0.77	0.26	0.14	0.06	0.11	0.74	0.15	0.01	0.01
	±%	−7	−2	−2	3	5	−26	13	−23	−16	10	−26
	SS	*∗*					*∗∗*		*∗∗*	*∗∗*		*∗∗*
375 (*n* = 9)	Mean	377.0	2.06	9.51	2.22	1.24	0.30	0.71	1.42	1.14	0.093	0.023
	SD	26.43	0.14	0.99	0.25	0.22	0.05	0.10	0.68	0.12	0.01	0.00
	±%	−11	−4	−5	−3	9	−31	1	−59	−30	20	−16
	SS	*∗∗*					*∗∗*		*∗∗*	*∗∗*	*∗*	

Females												
Control (*n* = 10)	Mean	241.9	1.94	6.63	1.61	0.83	0.41	0.55	0.66	0.165	0.088	0.021
	SD	15.55	0.07	0.70	0.21	0.07	0.06	0.11	0.09	0.036	0.012	0.003
180 (*n* = 10)	Mean	242.5	2.01	7.41	1.59	0.77	0.38	0.63	0.88	0.154	0.085	0.022
	SD	14.74	0.11	0.62	0.14	0.05	0.05	0.10	0.28	0.027	0.018	0.006
	±%	0	4	12	−1	−7	−8	15	32	−7	−4	9
	SS					*∗*			*∗*			
300 (*n* = 8)	Mean	250.4	1.98	7.07	1.56	0.92	0.33	0.61	0.58	0.148	0.092	0.022
	SD	15.97	0.08	0.56	0.09	0.09	0.05	0.06	0.18	0.034	0.016	0.004
	±%	4	2	7	−3	11	−20	11	−12	−10	4	6
	SS					*∗*	*∗∗*					
375 (*n* = 10)	Mean	247.3	1.96	7.16	1.55	0.95	0.31	0.61	0.57	0.151	0.095	0.022
	SD	21.06	0.06	0.84	0.14	0.14	0.07	0.11	0.19	0.031	0.016	0.005
	±%	2.2	0.7	8.0	−3.5	14.7	−25.0	11.1	−13.2	−8.3	6.9	8.3
	SS											

±%, percent deviation versus control, mg/kg bw/d, milligrams per kilogram body weight per day; SD, standard deviation; g, grams; SS, statistical significance; *n*, number of animals.

^*∗*^ *p* < 0.05; ^*∗∗*^ *p* < 0.01.

**Table 10 tab10:** Summary of organ weights relative to body weight (%).

Group (mg/kg bw/d)		Organ weight relative to body weight (%)									
		Brain	Liver	Kidneys	Heart	Thymus	Spleen	Testes or uterus	Epididymides or ovaries	Adrenals	Thyroids
Males											
Control (*n* = 10)	Mean	0.512	2.371	0.538	0.271	0.104	0.167	0.825	0.384	0.018	0.007
	SD	0.031	0.206	0.032	0.017	0.027	0.019	0.051	0.024	0.003	0.001
180 (*n* = 10)	Mean	0.519	2.551	0.564	0.269	0.096	0.183	0.831	0.391	0.018	0.005
	SD	0.021	0.137	0.034	0.037	0.018	0.011	0.060	0.031	0.003	0.001
	±%	1	8	5	−1	−8	10	1	2	−5	−19
	SS		*∗*				*∗*				*∗*
300 (*n* = 10)	Mean	0.540	2.502	0.593	0.305	0.082	0.203	0.692	0.347	0.022	0.005
	SD	0.054	0.099	0.045	0.031	0.011	0.021	0.201	0.046	0.003	0.001
	±%	5	6	10	13	−21	22	−16	−10	18	−20
	SS			*∗∗*	*∗*	*∗*	*∗∗*		*∗*	*∗*	*∗*
375 (*n* = 9)	Mean	0.549	2.519	0.588	0.329	0.079	0.189	0.379	0.304	0.025	0.006
	SD	0.040	0.148	0.043	0.047	0.009	0.019	0.187	0.040	0.003	0.001
	±%	7	6	9	22	−23	13	−54	−21	35	−5
	SS			*∗*	*∗∗*	*∗*	*∗*	*∗∗*	*∗∗*	*∗∗*	

Females											
Control (*n* = 10)	Mean	0.807	2.739	0.663	0.343	0.170	0.226	0.274	0.068	0.037	0.009
	SD	0.064	0.215	0.058	0.032	0.023	0.041	0.036	0.014	0.005	0.002
180 (*n* = 10)	Mean	0.831	3.051	0.656	0.316	0.155	0.260	0.359	0.064	0.035	0.009
	SD	0.040	0.110	0.049	0.023	0.017	0.034	0.107	0.013	0.007	0.002
	±%	3	11	−1	−8	−9	15	31	−6	−4	7
	SS		*∗∗*					*∗*			
300 (*n* = 8)	Mean	0.794	2.828	0.625	0.367	0.132	0.244	0.233	0.059	0.037	0.009
	SD	0.054	0.204	0.025	0.031	0.019	0.029	0.078	0.012	0.005	0.001
	±%	−2	3	−6	7	−23	8	−15	−13	0	2
	SS					*∗∗*					
375 (*n* = 10)	Mean	0.796	2.892	0.628	0.384	0.125	0.245	0.231	0.061	0.038	0.009
	SD	0.071	0.200	0.039	0.048	0.023	0.026	0.068	0.013	0.006	0.002
	±%	−1	6	−5	12	−27	8	−15	−10	5	5
	SS				*∗*	*∗∗*					

±%, percent deviation versus control, mg/kg bw/d, milligrams per kilogram body weight per day; SD, standard deviation; SS, statistical significance; *n*, number of animals.

^*∗*^ *p* < 0.05; ^*∗∗*^ *p* < 0.01.

**Table 11:** Summary of organ weight and body weight relative to brain weight (%).

Group (mg/kg bw/d)			Organ weight and body weight relative to brain weight (%)								
		Body weight	Liver	Kidneys	Heart	Thymus	Spleen	Testes or uterus	Epididymides or ovaries	Adrenals	Thyroids
Males											
Control (*n* = 10)	Mean	19608.6	465.49	105.54	52.99	19.90	32.71	161.64	75.55	3.58	1.29
	SD	1278.39	59.62	8.82	3.84	4.61	4.51	12.20	9.17	0.51	0.23
180 (*n* = 10)	Mean	19285.0	492.44	108.86	52.07	18.44	35.34	160.22	75.51	3.39	1.03
	SD	759.63	40.09	8.86	8.62	3.60	3.05	11.73	7.23	0.64	0.22
	±%	−1.7	6	3	−2	−7	8	−1	0	−5	−20
	SS										*∗*
300 (*n* = 10)	Mean	18700.4	467.55	110.87	56.86	15.45	37.96	127.31	64.29	4.04	0.97
	SD	1910.29	48.08	13.13	7.56	3.34	6.61	32.03	6.09	0.64	0.23
	±%	−5	0	5	7	−22	16	−21	−15	13	−25
	SS					*∗*		*∗*	*∗∗*		*∗∗*
375 (*n* = 9)	Mean	18314.2	460.80	107.34	60.39	14.59	34.75	68.41	55.41	4.52	1.14
	SD	1352.12	35.16	7.32	10.94	2.47	5.35	31.26	5.53	0.45	0.18
	±%	−7	−1	2	14	−27	6	−58	−27	26	−12
	SS					*∗∗*		*∗∗*	*∗∗*	*∗∗*	

Females											
Control (*n* = 10)	Mean	12466.2	341.455	82.693	42.583	21.197	28.267	34.109	8.473	4.554	1.056
	SD	957.28	37.492	10.797	3.797	2.917	5.757	5.119	1.847	0.652	0.183
180 (*n* = 10)	Mean	12062.5	368.27	78.98	38.14	18.71	31.46	43.10	7.66	4.20	1.10
	SD	591.84	25.74	4.94	3.23	2.17	4.83	12.09	1.39	0.78	0.23
	±%	−3	8	−4	−10	−12	11	26	−10	−8	4
	SS				*∗*			*∗*			
300 (*n* = 8)	Mean	12642.0	356.58	78.94	46.31	16.71	30.83	29.21	7.48	4.63	1.10
	SD	863.82	20.88	4.51	4.30	2.80	3.60	8.44	1.66	0.77	0.23
	±%	1	4	−5	9	−21	9	−14	−12	2	4
	SS					*∗∗*					
375 (*n* = 10)	Mean	12643.1	365.80	79.19	48.41	15.81	31.17	29.31	7.69	4.84	1.13
	SD	1076.51	40.84	6.37	6.57	3.62	5.39	9.33	1.45	0.85	0.25
	±%	1	7	−4	14	−25	10	−14	−9	6	7
	SS				*∗*	*∗∗*					

±%, percent deviation versus control, mg/kg bw/d, milligrams per kilogram body weight per day; SD, standard deviation; SS, statistical significance; *n*, number of animals.

^*∗*^ *p* < 0.05; ^*∗∗*^ *p* < 0.01.
